# Xenotropic Mouse Gammaretroviruses Isolated from Pre-Leukemic Tissues Include a Recombinant

**DOI:** 10.3390/v10080418

**Published:** 2018-08-09

**Authors:** Devinka Bamunusinghe, Matthew Skorski, Alicia Buckler-White, Christine A. Kozak

**Affiliations:** Laboratory of Molecular Microbiology, National Institute of Allergy and Infectious Diseases, Bethesda, MD 20892-0460, USA; devinka123@gmail.com (D.B.); mattskorski@gmail.com (M.S.); abwhite@niaid.nih.gov (A.B.-W.)

**Keywords:** recombinant retrovirus, xenotropic gammaretrovirus, endogenous retroviruses, retroviral origins

## Abstract

Naturally-occurring lymphomagenesis is induced by mouse leukemia viruses (MLVs) carried as endogenous retroviruses (ERVs). Replicating the ecotropic MLVs recombines with polytropic (P-ERVs) and xenotropic ERVs (X-ERVs) to generate pathogenic viruses with an altered host range. While most recovered nonecotropic recombinants have a polytropic host range, the X-MLVs are also present in the pre-leukemic tissues. We analyzed two such isolates from the AKR mice to identify their ERV progenitors and to look for evidence of recombination. AKR40 resembles the active X-ERV *Bxv1*, while AKR6 has a *Bxv1*-like backbone with substitutions that alter the long terminal repeat (LTR) enhancer and the envelope (*env)*. AKR6 has a modified xenotropic host range, and its Env residue changes all lie outside of the domain that governs the receptor choice. The AKR6 segment spanning the two substitutions, but not the entire AKR6 *env*-LTR, exists as an ERV, termed *Xmv67*, in AKR, but not in the C57BL/6 mice. This suggests that AKR6 is the product of one, not two, recombination events. *Xmv67* originated in the Asian mice. These data indicate that the recombinant X-MLVs that can be generated during lymphomagenesis, describe a novel X-ERV subtype found in the AKR genome, but not in the C57BL/6 reference genome, and identify residues in the envelope C-terminus that may influence the host range.

## 1. Introduction

Laboratory mice carry mouse leukemia viruses (MLVs) of three host range subgroups (reviewed in [1]). The MLVs with an ecotropic host range (E-MLVs) infect only the rodent cells, while the various xenotropic and polytropic MLVs (X-, P-MLVs, and collectively, X/P-MLVs) infect different subsets of the mouse taxa and other mammalian species [2,3]. The E-MLVs use the CAT-1 receptor [4], and all X/P-MLVs use the XPR1 receptor [5,6,7]. There are functionally distinct XPR1 variants in the genus *Mus*, but the majority of mouse strains carry *Xpr1^n^*, which allows for the infection of P-MLVs but not X-MLVs [8].

All three of the host range subgroups of the MLVs exist as infectious viruses and as endogenous retroviruses (ERVs), which are germline DNA copies acquired during past virus infections [9,10]. The specific ERVs can be present or absent in the mouse strains, and can be shared by the strains sharing a common ancestry. Many of the E-MLV ERVs (E-ERVs, termed *Emvs*) [9] and some of the X-ERVs (*Xmvs*) are capable of producing an infectious virus [1]. There are two subclasses of P-ERVs, the polytropic murine viruses (*Pmvs*) and modified polytropic murine viruses (*Mpmvs*) [11]. None of the infectious P-MLVs derive directly from *Pmvs* or *Mpmvs*, although these P-ERVs can contribute to the generation of intersubgroup recombinant viruses that have the distinctive P-MLV host range [12,13,14,15].

Recombinant P-MLVs are routinely generated de novo in laboratory mouse strains carrying replicating E-MLVs, and the appearance of these recombinants is linked to virus-induced lymphomas and leukemias [16,17,18]. High virus, high-leukemic mouse strains, like AKR, HRS, and C58, carry active *Emvs*, and the E-MLVs they generate can recombine with the products of nonecotropic ERVs to produce P-MLVs with an increased virulence and altered host range [18,19].

Our recent analyses of nonecotropic MLVs from leukemic and pre-leukemic tissues focused on the E-MLV derived P-MLVs [15], but the P-ERV substitutions have also been found in the lymphomagenic viruses from the mice infected with amphotropic MLVs [20], and there are multiple examples of intersubtype recombination involving *Xmvs*. For example, the melanoma associated MelARV MLV is a recombinant of the *Emv2* E-MLV and *Xmv45*/*XmvIV1* [21], the XMRV X-MLV is a recombinant of two nonecotropic ERV sequences [22], and some P-MLV recombinants can incorporate *Xmv-* and *Pmv*-derived sequences [15]. The analyses of the other infectious and endogenous MLVs of the inbred and wild mice suggest that some are recombinants that include X-MLV-related sequences [23,24].

Studies on the virological events that lead to naturally occurring leukemogenesis have largely been conducted in AKR mice [16,17,18]. Here, we sequenced two X-MLVs isolated from a pre-leukemic AKR mouse thymus, to identify their ERV progenitors and to determine if they had been subjected to recombination. One of these viruses, AKR6, is a recombinant derived from the *Bxv1 Xmv*, with substitutions derived from a distinctive ERV found in AKR, but not in the C57BL/6 (B6) reference genome. We demonstrated that this ERV subtype was acquired by Asian mice, and identified multiple envelope (Env) replacement mutations that may be responsible for the altered AKR6 xenotropic host range.

## 2. Materials and Methods

### 2.1. Viruses and Sequencing

The AKR6 and AKR40 X-MLVs were obtained from J. Hartley (National Institute of Allergy and Infectious Diseases, Bethesda, MD, USA) and had been isolated from the thymus tissue of two- and six-month old AKR mice, respectively [25]. The ferret MA139 cells, obtained from J. Hartley, were infected with AKR6, and were passaged several times. The viral genome was sequenced from the DNA of the infected ferret cells, using primers developed to sequence a set of P-MLVs [15].

The DNAs from various inbred mouse strains and wild-derived or wild-trapped mice were obtained from The Jackson Laboratory (Bar Harbor, ME, USA), the RIKEN BioResource Center through the National BioResource Project of the MEXT/AMED (Ibaraki, Japan), M. Potter (NCI, Bethesda, MD, USA), S. Rasheed (University of Southern California, Los Angeles, CA, USA), R. Abe (Naval Med. Res. Inst., Bethesda, MD, USA), S. Chattopadhyay, and H. Morse III (NIAID, Bethesda, MD, USA), or were isolated from the mice obtained from The Jackson Laboratory or from mice maintained in our laboratory (Table S1). To identify the AKR mouse ERVs related to AKR6, we used forward and reverse primers designed to amplify the AKR6 *env* and/or the long terminal repeat (LTR), as follows: F1, GAGCAGCATGGAAGGTCC; F2, CGGAATCCTCTATTGGAC; F3, CACTGCCCTAGTGACCACACG; R1, TGCAAACAGCAAAAGGCTTTATTGG; R2, AGTCTAACCCTCTTCGGT. Four primers were designed from AKR6; and F2 was based on the F1–R2 PCR product.

The AKR6 and AKR40 sequences were compared with previously described ERVs and MLVs. The highlighter plots were constructed using appropriate ERVs and MLVs (www.hiv.lanl.gov/content/sequence/HIGHLIGHT/highlighter_top.html) [26].

The sequences of AKR6 and AKR40 were deposited in GenBank under accession nos. MH450109-10. The 5′ 1.3 kb end of AKR6 *env* was previously sequenced (DQ199948) [27]. Three partial AKR6-related ERVs amplified from the AKR mouse DNA were deposited under MH443069-71.

### 2.2. Phylogenetic Trees

Phylogenetic trees were constructed in MEGA7 [28], using the maximum likelihood method based on the Kimura 2-parameter model with 500 bootstrap replicates [29]. Four trees were based on the two segments of AKR6 that diverge from *Bxv1,* the 5′ 357 bp of *env*, and the reverse transcriptase (RT) domain of *pol*. The alignments were generated by MUSCLE [30] and were corrected manually. The initial trees for the heuristic search were obtained automatically by applying neighbor-join and BioNJ algorithms to a matrix of pairwise distances estimated using the Maximum Composite Likelihood (MCL) approach, and then selecting the topology with a superior log likelihood value. A discrete Gamma distribution was used to model the evolutionary rate differences among the sites. All of the positions with less than a 95% site coverage were eliminated. That is, fewer than 5% alignment gaps, missing data, and ambiguous bases were allowed at any position. The trees included up to 14 *Xmvs* and representative *Pmvs* and *Mpmvs* from the B6 reference genome [10,31], as well as the following: *PreXMRV-1* (FR871849), *PreXMRV-2* (FR871850), NZB-9-1 (K02730, EU035300), CasE#1 (KU324802), Cz524 (KU324804), Kyushu (KU324806), CAST-X (KU324803), and a set of Y chromosome-linked ERVs that, together with the chromosome 5 *Xmv45*/*XmvIV1*, comprise the distinct *XmvIV* subset of *Xmvs* [21,23,32].

### 2.3. Inbred Strain Distribution and Subspecies Origins of AKR6-Related ERVs

We used the BLAST/BLAT tool [33] on the Ensembl website (https://useast.ensembl.org/Mus_musculus/Tools/Blast?db=core) [34] to search for the AKR6 related ERV sequences in the B6 mouse reference genome, and in the draft sequences of the AKR and 10 other mouse genomes generated by the Mouse Genomes Group at the Wellcome Trust Sanger Institute (https://www.sanger.ac.uk/science/data/mouse-genomes-project).

The DNA from 44 laboratory strains and from 43 wild-derived and wild-caught mice of the *M. musculus* subspecies were screened for the AKR6-related X-ERVs, with primers F2 and R1, and for a specific AKR6-related ERV found in AKR mice, *Xmv67*, using a primer from the viral LTR [35] and the flanking sequence primer, GGCTCTTGTCTGGCTTTAACC. 

The Mouse Phylogeny Viewer (MPV) (http://msub.csbio.unc.edu) [36] was used to determine the subspecies origins of the AKR6-related ERV found in AKR strain of mice. Because the NCBI37/mm9 B6 assembly was used to create the MPV database, we first determined the B6 coordinates for this ERV using cellular sequences flanking the ERV in the AKR genome to do a BLAT search of NCBI37/mm9 with the UCSC Genome browser (http://genome.ucsc.edu/). 

## 3. Results and Discussion

### 3.1. Sequence Analysis of Two X-MLVs

**Figure 1:**
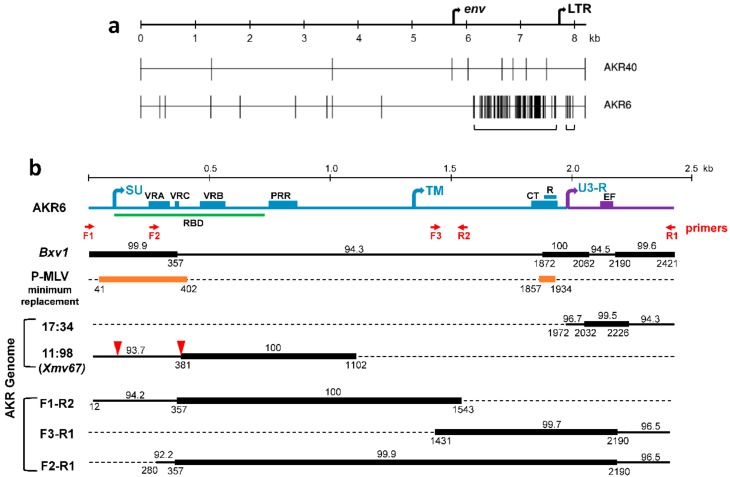
Comparative analysis of AKR6 and AKR40 X-MLVs. (**a**) Highlighter plots use vertical lines to identify nucleotide mismatches relative to the *Bxv1 Xmv*. Brackets indicate two substituted segments in AKR6 relative to *Bxv1*. (**b**) Sequence relationships between AKR6 and related endogenous retroviruses (ERVs) and PCR-amplified segments from AKR mice. The *env*-U3-R segment of the viral genome diagramed at the top notes the locations of the three variable domains (VRA, VRB, and VRC), the proline rich region (PRR) and the receptor binding domain (RBD) within the surface (SU) domain of Env, the cytoplasmic tail (CT) and R-peptide (R) in the transmembrane (TM) domain, and the enhancer framework (EF) within U3. Six black lines show segmental sequence comparisons to AKR6 for *Bxv1* and 5 AKR-derived AKR6-related sequences, two from the AKR genome representing a solo long terminal repeat (LTR) and a partial *env* with two duplications associated with sequencing gaps (red triangles), and three PCR products from AKR amplified using the indicated primers (in red). The orange line positions the two replacements common to the P-MLVs. The numbers above each line show % identity to AKR6, and the numbers below the line identify the start and stop positions of each segment relative to AKR6. Identity > 99% is shown with a thick line.

The AKR6 and AKR40 MLV isolates both show a xenotropic host range, in that they can infect mink cells, but fail to infect laboratory mouse cells [37]. An analysis of their full-length genomes shows that, unlike the P-MLVs isolated from these tissues, neither of the X-MLVs incorporate the E-MLV-derived sequences. Highlighter plots show the genome-wide distribution of the nucleotide differences between the two sequenced X-MLVs and their closest homolog, the *Bxv1 Xmv* (Figure 1a). *Bxv1* is a full-length non-defective ERV that is normally quiescent, but can be induced to produce an infectious virus [38]; *Bxv1* was first identified in B6, but is also present in the AKR genome [10,39]. The AKR40 genome is 99.9% identical to *Bxv1.* While AKR6 shows a comparably high homology to *Bxv1* throughout most of its genome, there are two segments of 129 bp and 1.5 kb that show a reduced identity to *Bxv1*, of 94.3% and 94.5%, respectively (Figure 1b); the intervening 191 bp are 100% identical to *Bxv1*. The 129 bp replacement is in the LTR, and the 1.5 kb replacement is in *env*.

**Figure 2:**
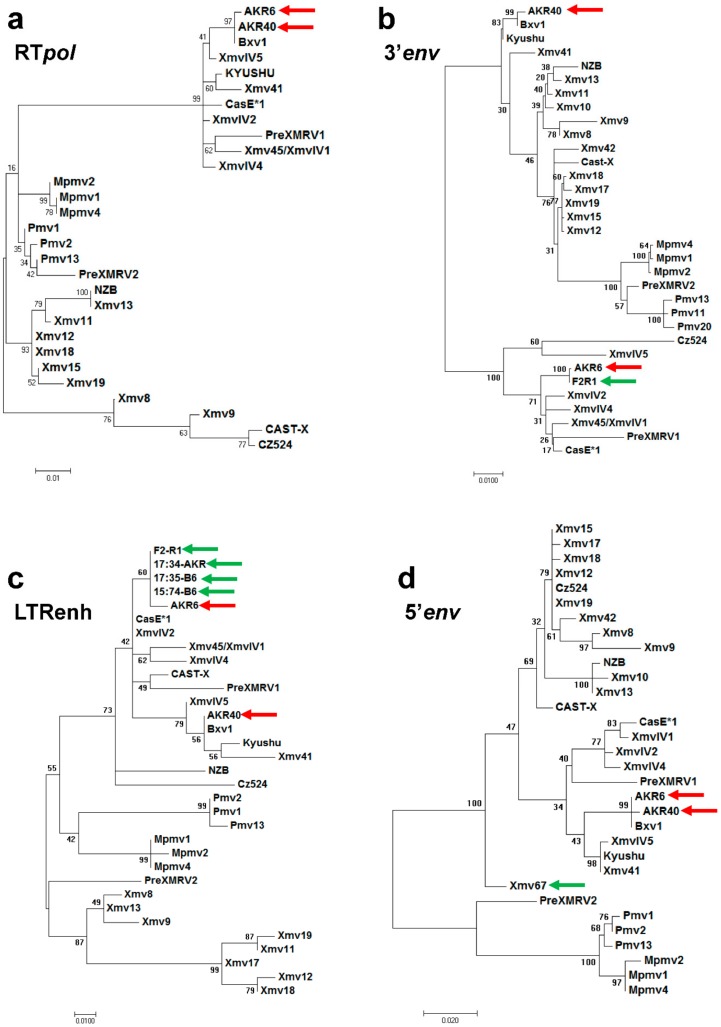
Phylogenetic trees of four domains of the MLV genome. (**a**) RT*pol*, (**b**,**c**) the *env* and U3-LTR enhancer substitutions in AKR6, (**d**) the 5’ VRA-containing end of *env*. The percentage of trees in which the associated taxa clustered together is shown next to the branches. The trees are drawn to scale, with the branch lengths measured in the number of substitutions per site. Red arrows identify AKR40 and AKR6 MLVs, and green arrows identify AKR6-related PCR products or mined sequences from the AKR/J genome. All of the *Xmvs*, *Pmvs*, and *Mpmvs* are from the B6 reference genome.

Four phylogenetic trees were constructed to compare the AKR6 to the previously described X/P-MLVs and ERVs, based on RT*pol* (Figure 2a)*,* the AKR6 1.5 kb and 129 bp replacements (Figure 2b,c), as well as the *env* segment upstream of the AKR6 replacement (Figure 2d). While the B6 polytropic *Pmvs* and *Mpmvs* cluster together in all four trees, the B6 *Xmvs* show much more sequence variation, as previously shown for their full length genomes [31]. In all four trees, AKR40 clusters with *Bxv1.* AKR6 also clusters with *Bxv1* in the RT*pol* and 5’*env* trees (Figure 2a,d), but the AKR6 *env* substitution groups with the *XmvIV* subset of the *Xmv* ERVs [23,32] (Figure 2b,c). BLAST searches confirm that the best sequence matches for the AKR6 *env* replacement in the reference genome are among the *XmvIV* ERVs, with ~98% identity. The *XmvIV* ERVs do not produce an infectious virus, but related segments have been found in other recombinant viruses from inbred mice, including the melanoma-associated MLV, MelARV, and several P-MLVs as well as wild mouse MLVs [15,21,23].

The 129 bp LTR substitution shows closest identity to two solo LTRs in the B6 reference genome (Chr15:74933718-74934248; 17:35000213-35000742) with a 99.4% identity to both (Figure 2c). The high sequence identity is, however, restricted to this 129 bp segment, as the identity at the 3′ and 5′ ends of these LTRs is reduced to 96.7% and 94.3%, respectively (Figure 1b).

Both replacements in the AKR6 genome alter the functionally important viral segments. The smaller 129 bp replacement spans the U3 LTR enhancer segment containing the four transcription factor binding sites, which form the retroviral enhancer framework, namely, LVb, core, NF1, and GRE [40]. The generation of virulent P-MLVs is associated with alterations in two enhancer elements, core and NF1, and these changes in the P-MLVs can result from mutation or recombination [15]. For AKR6, however, the enhancer replacement introduces the more benign variant of the core site (TGTGGTCAA vs. TGTGGTCGA) [41], without changing the other sites.

The larger 1.5 kb AKR6 replacement in *env* begins in the middle of the receptor binding domain of *env* (RBD*env),* extending from the end of the VRC variable region to the beginning of the R peptide in the cytoplasmic tail of the transmembrane subunit of *env* (TM*env*) (Figure 1b). Our previous analysis of 16 P-MLVs showed that these viruses, derived from the recombination of E-MLVs and P-ERVs, showed 16 different recombination patterns that collectively covered the whole viral genome. All 16 shared two and only two common replacements, both in *env*, at the 5′ end of the surface (SU), and the 3′ cytoplasmic tail R peptide (Figure 1b) [15]. These two segments show a substantial sequence divergence between the E-MLV backbone of these P-MLVs and their P-ERV-derived substitutions. The replicating viruses carry either both P-MLV segments or both E-MLV types, but never a combination, suggesting a sequence-based constraint. Compared to these P-MLVs, AKR6 shows a reciprocal replacement pattern in that the two *env* segments that are replaced in all of the P-MLVs are retained by AKR6 from its *Bxv1* progenitor, while the intervening sequence is replaced (Figure 1b). The R peptide has been implicated in the regulation of the Env ectodomain [42], so the fact that these two *env* segments are either coordinately retained (in AKR6) or replaced (in P-MLVs), suggests restrictions on the pairing of some variants of these two *env* segments. The maintenance of both *Bxv1*-like *env* segments in the AKR6 recombinant suggests that the comparable segments in its ERV progenitor either produce nonfunctional proteins in either or both of these segments, or that these segments encode variants that are not compatible with either of the *Bxv1 env* segments.

### 3.2. ERV Progenitors of the AKR6 Replacements

We used in silico searches and PCR to look for possible progenitors of AKR6 in the AKR mouse genome (Figure 1b). Firstly, we screened the available AKR genome draft sequence using BLAST/BLAT. The only good match for the LTR enhancer replacement, at 17:34443814-34444343, is a solo LTR that is orthologous to one also found in the B6 genome, as described above. Like its B6 ortholog, this AKR LTR shows 100% homology to the enhancer region of the AKR6 LTR, but has only 96.7% and 94.3% identity to the 5′ and 3′ ends of this LTR.

We also identified a single sequence in the AKR draft genome related to the *env* replacement (11:98208845-98210232). This partial ERV has no ortholog in B6 and was not identified among a set of AKR *Xmvs* [10]. Therefore, we gave it the next consecutive *Xmv* number, *Xmv67*. In this AKR assembly, *Xmv67* is flanked by large sequencing gaps and contains two internal gaps, both of which are associated with duplications (Figure 1b). The 3′ 721 bp of this sequence is identical to the corresponding AKR6 *env* segment, but the sequence at its 5′ end is more divergent, with only a 93.7% identity to AKR6. This ERV segment encodes an *env* ORF, but is distinct from the other X-ERVs (Figure 2d).

Next, we coupled AKR6- and *Xmv*-specific primers to amplify the extended segments containing the AKR6-related *env* and/or LTR replacement segment from the AKR genome. We consistently identified the same overlapping amplicons (Figure 1b). F1–R2 contains the 5′ end of the *env* replacement segment, and F3-R1 contains much of the AKR6 TM*env*, the LTR substitution, and the intervening sequence, which is shared with *Bxv1*. The 5′ end of F1–R2, and the 3′ end of F3–R1, are not found in the AKR6 viral genome (Figure 1b), nor are they closely related to the B6 *Xmvs* (shown for 5′ *env*, Figure 2d). A primer designed from the divergent segment at the 5′ end of F1–R2 was used to generate an extended amplicon, F2–R1, produced by AKR but not B6. The segment from 357-2190 of F2–R1 that is identical to AKR6, includes the two segments, in *env* and LTR, that distinguish AKR6 from *Bxv1*, as well as the intervening *Bxv1*-like segment. The presence of all three of these segments in this single ERV-derived PCR product indicates that the AKR6 substitution spanning the *env* and LTR exists as an ERV in AKR, but is not found in the B6 reference genome. Thus, AKR6 is a recombinant likely derived from one, not two, recombination events, and furthermore, the sequence differences between AKR6 and *Xmv67* at the 5′ and 3′ ends, show that AKR6 does not exist as an intact ERV in AKR.

### 3.3. Acquisition of AKR6-Related ERVs in M. musculus Subspecies

**Figure 3:**
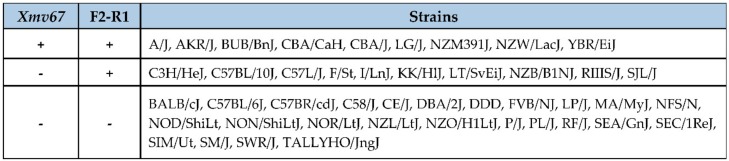
Distribution of *Xmv67* and F2–R1 in common inbred mouse strains.

The classical inbred strains of the laboratory mouse are mosaics of 3 *M. musculus* subspecies: *domesticus*, *musculus*, and *castaneus* [43]. *Xmvs* and the single autosomal copy of the *XmvIV* subgroup can be traced to their Asian *M. m. musculus* and *M. m. castaneus* progenitors [23,35]. To determine if the *Xmv67* AKR6 progenitor has a similar wild mouse origin or is a recent acquisition, we designed a primer from the flanking nonviral sequences downstream of *Xmv67*, and typed a set of mouse DNA for a cell-virus junction product specific to *Xmv67*. The strain distribution of this ERV (Figure 3) does not correspond to that of any of the previously described ERVs identified in AKR [10]. The inbred strains were also typed for the AKR6-related F2–R1 internal viral fragment (Figure 3); this sequence is found in ten strains that lack the *Xmv67* insertion, indicating that F2–R1 identifies a distinctive subset of *Xmvs* found in some inbred strains.

**Figure 4:**
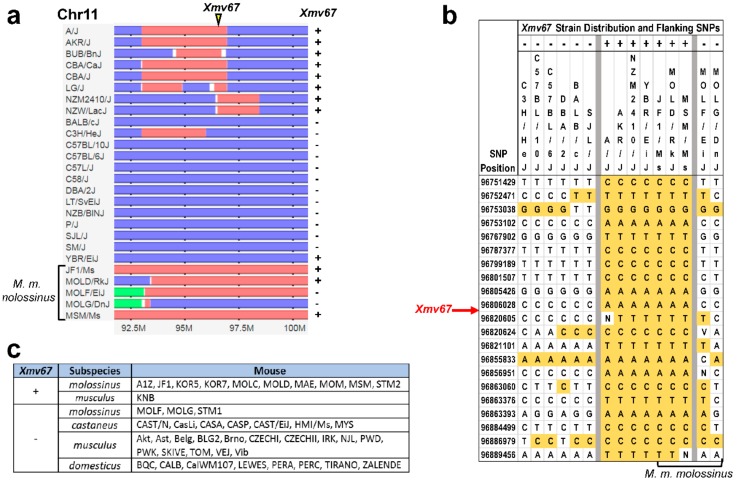
ERVs related to the AKR6 substitutions map to chromosomal regions that originated in Asian mice. (**a**) Horizontal tracks represent a 10-Mb segment of chromosome 11 in selected strains and subspecies typed for *Xmv67*. Chromosomal regions originating from *M. m. domesticus* are in blue and *M. m. musculus* in red. PCR typing is shown on the right. The position of the AKR ERV insertion site at Chr11:97 (based on NCBI37/mm9) is marked by an inverted triangle. (**b**) Polymorphic SNPs surrounding the *Xmv67* insertion site are shown for a subset of the tested mice. Yellow highlights the AKR-specific SNPs. (**c**) Distribution of *Xmv67* in subspecies of *M. musculus*.

Of the 44 strains typed for *Xmv67*, 39 are included in the MPV database, which used single nucleotide polymorphisms (SNPs) to infer the subspecific origins of DNA segments along each chromosome for 100 inbred strains [44]. Figure 4a shows a 10 Mb segment containing the *Xmv67* insertion site. This site lies in a genomic segment of AKR that originated in *M. m. musculus,* and this site is also clearly *M. m. musculus*-derived in seven of the eight other *Xmv67*-positive strains, but in none of the negative strains. This ERV is also detected in three of the four wild-derived Japanese mice that have *M. m. musculus*-derived sequences at this locus (Figure 4a).

Figure 4a shows two discrepancies, the inbred strain, YBR, which places its *Xmv67* locus in a *M. m. domesticus*-derived segment, and the wild-derived strain, MOLF, which lacks *Xmv67* but is *M. m*. *musculus* in this chromosomal segment. Because small segments of intersubspecies recombination can be missed, we examined the SNP polymorphisms around this integration site, and showed that YBR but not MOLF shares the SNPs flanking the ERV insertion site that are exclusive to the *Xmv67*-positive mice (Figure 4b).

The Asian mouse origin of *Xmv67* was confirmed by PCR-typing of the DNA from the *M. musculus* subspecies (Figure 4c)*. Xmv67* was identified in 10 of the 13 *M. m. molossinus*, the Japanese mouse that is a natural hybrid of *M. m. musculus* and *M. m. castaneus*, in 1 of the 16 *M. m. musculus*; and in none of the 7 *M. m. castaneus* and 8 *M. m. domesticus*. These data indicate that *Xmv67*, like all other X-ERVs, originated in Asian mice, but unlike the other *Xmvs,* some of which are found in *castaneus* as well as *molossinus*, *Xmv67* may derive from the *musculus* ancestor of *molossinus*.

### 3.4. Env Replacement Mutations Linked to the Modified AKR6 Host Range

**Figure 5:**
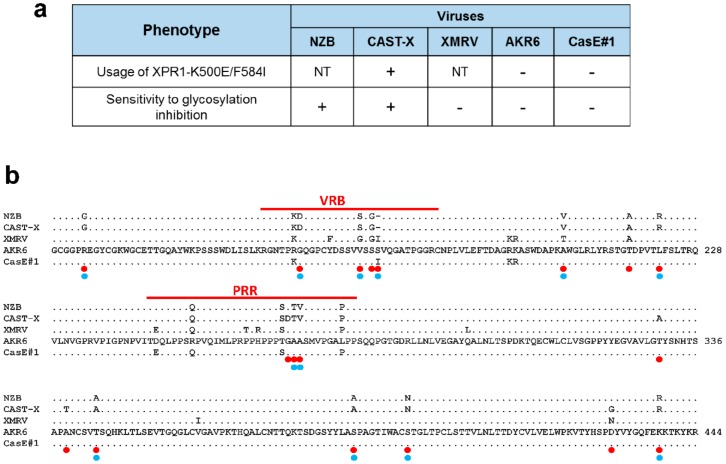
Multiple replacement mutations alter the C-terminal end of the AKR6 SU Env. (**a**) AKR6 host range differs from other X-MLVs [35,45,46]. (**b**) Alignment of the amino acid sequences in the replaced segment of the AKR6 Env compared to X-MLVs with shared or different host range properties. Red lines indicate the variable VRB and PRR domains. Red dots mark sites that distinguish viruses able to use a mutant XPR1 receptor and blue dots mark viruses that differ in their sensitivity to glycosylation inhibition.

The AKR6 X-MLV was initially described as a representative X-MLV in host range [37], but this virus shows differences in entry-related phenotypes compared with other X-MLVs (Figure 5a). Firstly, AKR6 and one other X/P-MLV, CasE#1, are poorly infectious for an XPR1 receptor with the double mutation K500E/F584I [35]. Secondly, the AKR6, CasE#1, and XMRV MLVs uniquely fail to infect the Chinese hamster cells in which the glycosylation is inhibited [45,46]. These previous studies did not attempt to identify the Env residues responsible for these host range differences.

AKR6 is identical to *Bxv1* in the N-terminus of Env containing VRA, which determines the receptor choice [47]. The AKR6 substitution begins in VRC and spans the rest of SU*env* including the variant segments VRB and the proline rich region (PRR). A comparison of the *env* sequences of the relevant X-MLVs identified shared replacements at 18 sites for the viruses that differentially infect XPR1 K500E/F584I, 12 of which also distinguished viruses that vary in sensitivity to glycosylation inhibition in hamster cells (Figure 5b). Four of these sites map to VRB and three adjacent sites are in PRR; previous studies on MLVs and other gammaretroviruses implicate both VRB and PRR in receptor interactions [47,48]. The remaining replacement mutations are scattered, but one site implicated in the mutant receptor usage, T217A, is at one of two adjacent sites in the RBD C-terminus, previously shown to permit some X/P-MLVs to infect human cells [49]. This analysis identifies multiple sites in the C-terminal end of the RBD that may be responsible for the altered receptor properties of AKR6 and other MLVs.

## 4. Conclusions

We show that the recombinant MLVs generated during pathogenesis can include X-MLVs, and that intrasubtype recombination can modify their phenotype, in this case viral tropism. The substitution in AKR6 that extends from the VRC of RBD*env* through the LTR enhancer, derives from an *Xmv* ERV variant found in AKR, but not B6 mice. This ERV substitution is most closely related to, but not identical to, the *XmvIV* subgroup of *Xmvs* and, like all other *Xmvs*, is traceable to the ERVs present in Japanese mice, known contributors to the fancy mouse colonies that gave rise to the classical laboratory strains. 

The inbred strains differ in their MLV ERV complements, largely due to the different insertions of the same set of MLV subtypes [10,31]. The AKR6 *env*-LTR ERV progenitor is a novel X-ERV subtype present in at least one copy in AKR, but most other laboratory strains lack this ERV. The only closely related ERVs retained in the B6 reference genome are represented by two solo LTRs. The 5’ end of the AKR *Xmv67* Env is distinctive, as are segments of the AKR6-related LTRs, and that these sequences are not found in replicating viruses suggests they may not be functional.

The presence of the *Xmv*-derived viruses in the AKR pre-leukemic thymus is not surprising. While the *Bxv1 Xmv* is normally quiescent, its expression can be induced by immunological stimulation [50], and the virus spread of X/P-MLVs is not strictly governed by the receptor compatibility. The greater abundance of recombinant P-MLVs in preleukemic tissues may be due to the fact that these viruses have three alternative methods of spread, they can use the AKR XPR1 receptor, they are packaged preferentially into E-MLV virions that rely on the CAT-1 receptor for entry [51], and they can also use the CAT-1 receptor if soluble E-MLV SU-Env is present (transactivation) [52]. X-MLVs, on the other hand, cannot use the AKR XPR1 receptor and are not efficiently transactivated by the E-MLV SU-Env [52], but the X-MLV genomes can be co-packaged with other MLV subtypes, resulting in P-MLVs that incorporate X-MLV sequences [15] or, as shown there, can also produce recombinant X-MLVs like AKR6. The routine generation of recombinant X-MLVs and P-MLVs that include *Xmv*-derived segments indicates that these recombinants are a common event that may have biological consequences during virus-induced leukemogenesis.

## Supplementary Materials

The following are available online at http://www.mdpi.com/1999-4915/10/8/418/s1, Table S1: Wild-derived or wild-caught *M. musculus* mice typed for *Xmv67*.
