# Supplementary material for: Xenotropic Mouse Gammaretroviruses Isolated from Pre-Leukemic Tissues Include a Recombinant
Source: Viruses. 2018 Aug 9;10(8):418. doi: 10.3390/v10080418 (PMC6116186; doi:10.3390/v10080418)
Supplement: Supplementary file 1 [file viruses-10-00418-s001.zip › Table S1 final.docx]

Table S1. Wild-derived or wild-caught *M. musculus* mice typed for *Xmv67*.

| **Subspecies** | **Previous species name or other designation** | **Geographic location** | **Source** |
| --- | --- | --- | --- |
| *castaneus* | CASA/RkJ | Thonburi, Thailand | Jackson Laboratory |
| *castaneus* | CAST/N | Thaila Thonburi, Thailand | Potter |
| *castaneus* | CAST/EiJ | Thonburi, Thailand | Jackson Laboratory |
| *castaneus* | CAS/Li | Thonburi, Thailand | Potter |
| *castaneus* | CASP/1Nga | Los Banos, Philippines | RIKEN |
| *castaneus* | HMI/Ms | Hemei,Taiwan | RIKEN |
| *castaneus* | MYS/Mz |  | RIKEN |
| *molossinus* | JF1 | Fancy mouse | Jackson Laboratory |
| *molossinus* | MOLD/RkJ, MOLF/EiJ, MOLG/DnJ, MOLC/Rk | Fukuoka, Kyushu, Japan | Jackson Laboratory |
| *molossinus* | MSM/Ms | Mishima Prefecture, Japan | Jackson Laboratory |
| *molossinus* | STM1, STM2 |  | RIKEN |
| *molossinus* | KOR5, KOR7 | Koriyama, Fukushima, Japan | RIKEN |
| *molossinus* | AIZ | Aizu, Japan | RIKEN |
| *molossinus* | MAE | Maesawa, Iwate, Japan | RIKEN |
| *molossinus* | MOM | Mizuho Aichi, Japan | RIKEN |
| *musculus* | Skive | Skive, Denmark | Potter |
| *musculus* | CzI, CzI-ochre, CZECHI/EiJ | Morovia, Czech Republic | Potter, Jackson Laboratory |
| *musculus* | CzII, CZECHII/EiJ | Bratislava, Slovakia | Potter, Jackson Laboratory |
| *musculus* | PWD/PhJ | Kunratice, Czech Republic | Jackson Laboratory |
| *musculus* | PWK/PhJ | Lhotka, Czech Republic | Jackson Laboratory |
| *musculus* | VEJ | Vejrumbro, Denmark | Potter, Chattopadhyay/Morse |
| *musculus* | IRK/Tua | Irkutsk, Russia | RIKEN |
| *musculus* | TOM/Tua | Tomsk, Russia | RIKEN |
| *musculus* | KNB/Tua (*wagneri*) | Balkhash Lake, Kazakhstan | RIKEN |
| *musculus* | NJL | Northern Jutland, Denmark | RIKEN |
| *musculus* | Akt/TUA | Aktyubinsk, Kazakhstan | RIKEN |
| *musculus* | Ast/TUA (*wagneri*) | Astrakhan, Russia | RIKEN |
| *musculus* | BLG2 | Toshevo, Bulgaria | Abe |
| *musculus* | Brno | Brno, Czech Republic | Chattopadhyay, Morse |
| *musculus* | Belg | Belgrade, Yugoslavia | Chattopadhyay, Morse |
| *musculus* | Vib | Viborg, Denmark | Chattopadhyay, Morse |
| *domesticus* | LW, LEWES/EiJ | Lewes, Delaware | Potter, Jackson Laboratory |
| *domesticus* | CalWM107 | Lake Casitas, California | Rasheed |
| *domesticus* | BQC | Bouquet Canyon, California | Potter |
| *domesticus* | CALB/RkJ | California | Jackson Laboratory |
| *domesticus* | PERA/EiJ, PERC/EiJ | Rimac Valley, Peru | Jackson Laboratory |
| *domesticus* | *poschiavinus* (Posch-1), TIRANO | Tirano, Italy | Potter |
| *domesticus* | *poschiavinus* (Posch-2), ZALENDE | Zalende, Switzerland | Potter |
